# Efficacy and Cardiotoxicity of Liposomal Doxorubicin-Based Chemotherapy in Advanced Breast Cancer: A Meta-Analysis of Ten Randomized Controlled Trials

**DOI:** 10.1371/journal.pone.0133569

**Published:** 2015-07-23

**Authors:** Meiyuan Xing, Feifei Yan, Sufen Yu, Peng Shen

**Affiliations:** 1 Department of Library, the First Affiliated Hospital, College of Medicine, Zhejiang University, Zhejiang, China; 2 Department of Oncology, the First Affiliated Hospital, College of Medicine, Zhejiang University, Zhejiang, China; Seoul National University, REPUBLIC OF KOREA

## Abstract

**Background:**

Various trials have compared the efficacy and toxicity of liposomal doxorubicin-based chemotherapy with the conventional formulation of doxorubicin although arriving at inconsistent conclusions. To derive a conclusive assessment of the efficacy and cardiotoxicity associated with chemotherapy, we performed a meta-analysis by combining data from all eligible randomized controlled trials.

**Methods:**

We used the PubMed database to identify relevant studies published through December 28, 2014. Eligible studies included randomized controlled trials directly comparing the efficacy and cardiotoxicity of liposomal doxorubicin-based chemotherapy with conventional doxorubicin in advanced breast cancer with adequate data. Odds ratios (ORs) or hazard ratios (HRs) with 95% confidence intervals (CIs) were used to assess the efficacy and cardiotoxicity in a fixed-effects or random-effects model.

**Results:**

Ten randomized controlled trials containing efficacy and data from a total of 2,889 advanced breast cancer patients were included in this report. Liposomal doxorubicin-based chemotherapy was associated with a significant reduction in the risk of cardiotoxicity (OR = 0.46, 95% CI 0.23 to 0.92, p = 0.03) and a significant improvement in the overall response rate (ORR) (OR = 1.25, 95% CI 1.02 to 1.52, p=0.03) compared with conventional doxorubicin. An apparent improvement in progression-free survival (PFS) for patients treated with liposomal doxorubicin-based chemotherapy was noted; however, this difference was not significant (HR = 1.14, 95% CI 0.96 to 1.34, p = 0.12). In terms of overall survival (OS), no significant difference between the two chemotherapy regimens was noted (HR = 1.00, 95% CI 0.91 to 1.10, p = 0.93).

**Conclusion:**

The results of this meta-analysis suggest that liposomal doxorubicin-based chemotherapy is associated with a significant improvement in the ORR and a significant reduction in the risk of cardiotoxicity.

## Introduction

Breast cancer is the most frequent invasive cancer in women all over the world and the second highest cause of cancer death, after lung cancer [1]. Advanced breast cancer, including relapsed and metastatic breast cancer, remains incurable, and the therapeutic goals are palliating symptoms, delaying disease progression and prolonging OS time without negatively impacting the quality of life.

Anthracycline is one of the most effective agents for both early and advanced breast cancer [2]. However, the potential benefits of conventional anthracyclines are limited by the risk of cardiotoxicity, which is clearly related to cumulative dose [3–5]. Liposomal doxorubicin has been developed to reduce cardiotoxicity while preserving the antitumor efficacy [6].

Various non-comparative trials have demonstrated that liposomal doxorubicin was effective as a single agent or in combination with other drugs for the treatment of either anthracycline-treated or naive metastatic breast cancer patients [7–9]. Some trials demonstrated that liposomal doxorubicin reduced cardiotoxicity and had a similar antitumor efficacy compared with conventional anthracycline [10,11]. Additionally, some studies have shown that liposomal doxorubicin did not increase cardiotoxicity compared with anthracycline-free chemotherapy [12], whereas other trials indicated that liposomal doxorubicin was more effective than conventional anthracyclines with a similar cardiotoxicity [13].

Thus, it is important to conduct a meta-analysis addressing pertinent evidence to evaluate whether liposomal doxorubicin leads to lower cardiotoxicity while maintaining antitumor efficacy compared to other regimens. This report focused on cardiotoxicity, response, PFS and OS.

## Materials and Methods

### Search criteria

PubMed was searched for articles published from the earliest record to April 2015. Boolean operators were used as follows: (liposom* and doxorubicin OR DOX-SL OR Lipodox OR Doxil OR Caelyx OR Lipo-Dox OR DaunoXome) AND (breast tumor OR mammary neoplasm OR human mammary neoplasms OR mammary carcinoma OR human mammary carcinoma OR breast cancer OR cancer of breast OR mammary cancer OR breast carcinoma OR mammary adenocarcinoma OR BC OR breast neoplasms OR Breast Neoplasm) with no restriction on publication year or language. Manual searches of reference lists were performed to detect other reports not identified by our original search. This meta-analysis was performed in accordance with the Preferred Reporting Items for Meta-Analyses (PRISMA) statement checklist (S1 Checklist).

### Criteria for selection of published reports for meta-analysis

We reviewed the titles and abstracts of the identified articles, and included prospective randomized controlled trials that directly compared the efficacy and safety of liposomal doxorubicin with other agents as either a monotherapy or in combination in advanced breast cancer patients.

### Data extraction

Two authors independently extracted data using a standardized data collection form. The extracted data from each study included: the first author, year published, trial phase, patient characteristics, line of treatment, chemotherapeutic regimens, number of patients participating, and the main outcomes consisting of cardiotoxicity (defined by significant LVEF (left ventricular ejection fraction) changes), overall response (complete response + partial response), OS and PFS. If data were not reported in the original article or not displayed in the table, we extrapolated them from the accompanying graphs. We also attempted to contact the corresponding authors of eligible trials to obtain any further useful data for our analysis. When the two authors had disagreements, one or more additional author (s) joined the discussion until a consensus was achieved.

### Quality assessment

The 12-item scale, containing: randomised adequately, allocation concealed, patient blinded, care provider blinded, outcome assessor blinded, acceptable drop-out rate, ITT analysis, avoided selective reporting, similar baseline, similar or avoided cofactor, patient compliance and similar timing, was used to estimate the methodological quality of each trial[14].

### Statistical analysis

All data analyses were conducted with RevMan 5.0 analysis software (The Cochrane Collaboration, Copenhagen, Denmark). ORs and 95% CIs were used for the analysis of dichotomous outcomes. The generic inverse variance method was used to analyze HRs. The method reported by Parmar MK et al. was used to extract estimates of the log HR and its variance if this information was not provided clearly [15]. A chi-square test and I^2^ test were used to calculate the statistical heterogeneity. We considered I^2^ values of 25%, 50% and 75% as low, medium and high heterogeneity, respectively. If I^2^ < 50%, we used the fixed-effects model; otherwise, the random-effects model was used. We performed sensitivity analyses only if there were three or more studies included in the comparison. The influence of each single study on the results was evaluated by removing each study from consideration one at a time. Publication bias was assessed using funnel plots method.

## Results

**Fig 1:**
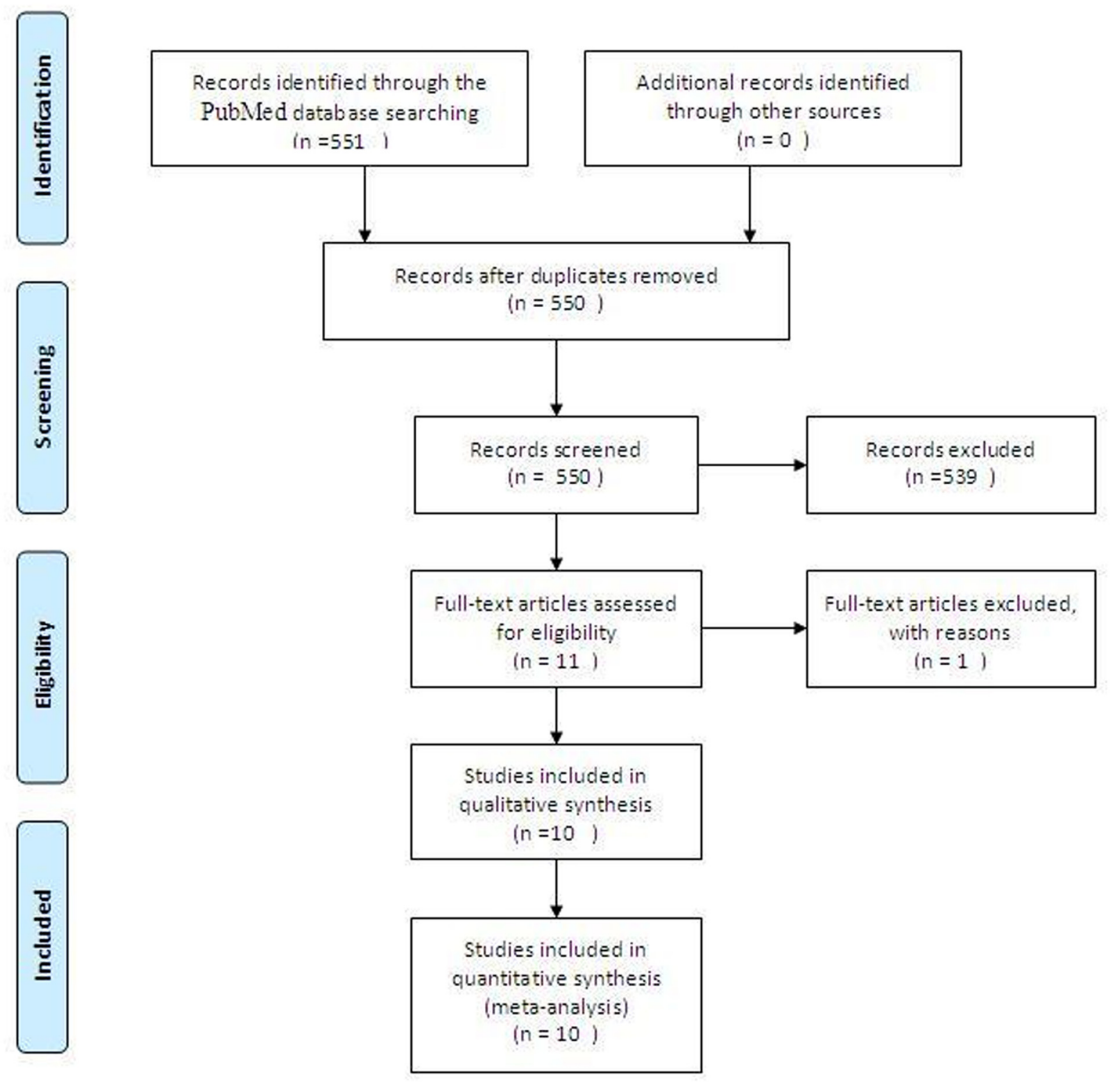
Flow Diagram for the Selection of Studies.

A literature search initially yielded 551 relevant citations. After the titles and abstracts were reviewed, only eleven articles met the criteria for inclusion in the report. One study [16] without eligible data regarding outcomes was excluded. Finally, ten studies [10–13,17–22] meeting the predetermined eligibility criteria were included in this meta-analysis. The study selection process is presented in Fig 1. They were all high-quality studies (S1 Table).

### Characteristics of included studies

The characteristics of the ten studies are presented in Tab 1. We identified eight phase III trials and two phase II trials with a total of 2,889 advanced breast cancer patients for this meta-analysis. These studies were all prospective randomized controlled trials. All patients in the eight trials presented with metastatic breast cancer, and all relapsed breast cancer patients were contained in one trial. One trial exclusively included taxane-refractory patients [18]. Five trials [10,11,13,19,20] compared liposomal doxorubicin-based chemotherapy with conventional-anthracycline-based chemotherapy. In the remaining five trials [12,17,18,21,22] liposomal doxorubicin-based chemotherapy was compared with anthracycline-free chemotherapy. (S2 Table).

### Efficacy of liposomal doxorubicin-based chemotherapy versus other chemotherapies

Fixed-effects models were used to determine the ORR and OS for low heterogeneity due to low heterogeneity (I^2^ = 0% and 7%, respectively). For PFS, a random-effects model was chosen given the high amount of heterogeneity (I^2^ = 69%, p = 0.002). Given that rare events were observed in one or more clinical trials, an OR model was used for the ORR.

**Fig 2:**
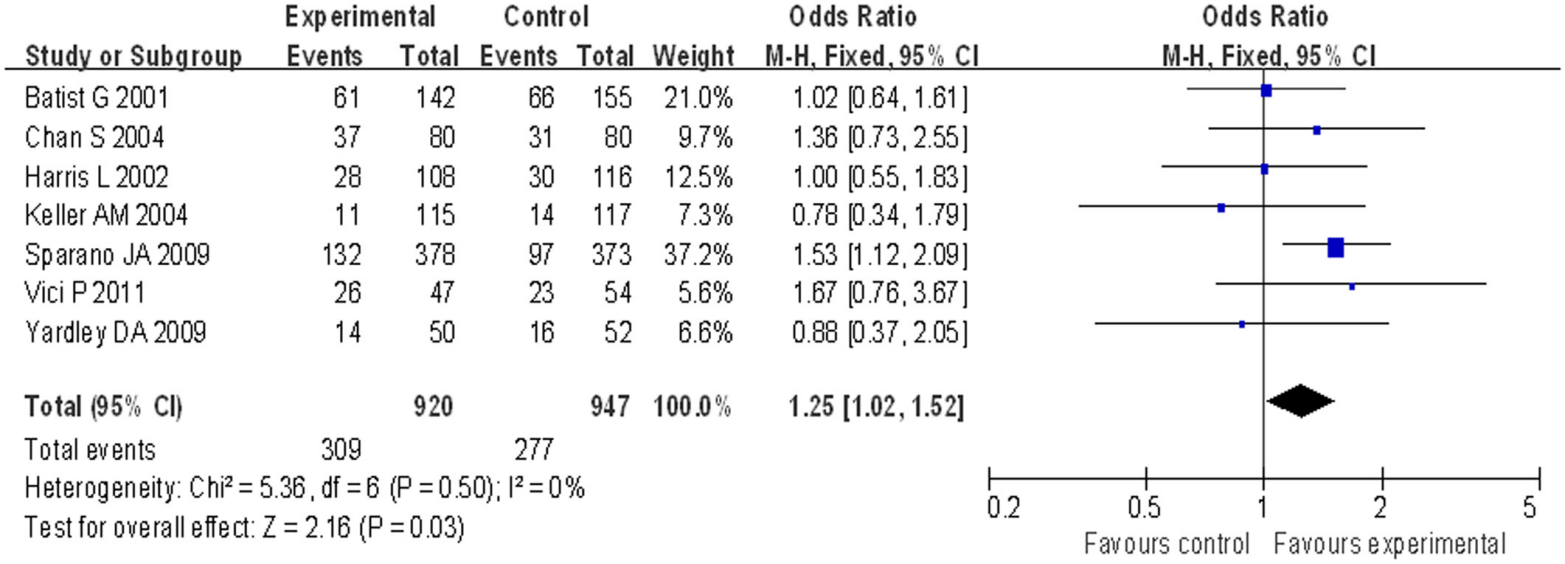
Forest plot of ORR comparison between two groups.

**Fig 3:**
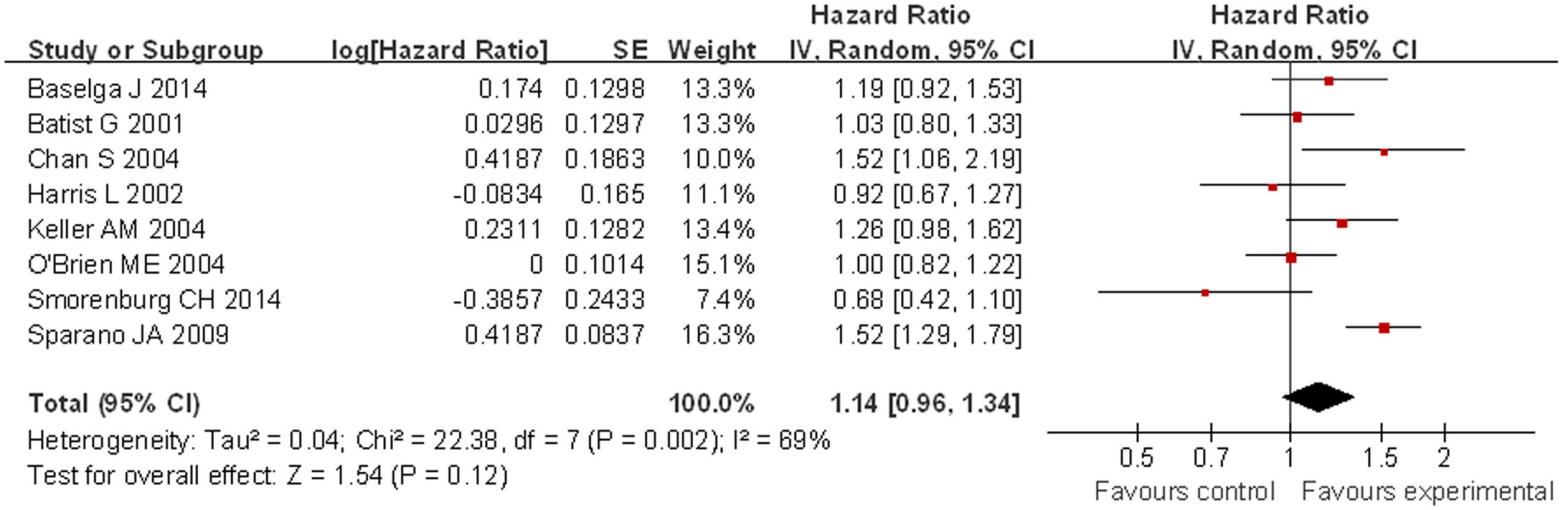
Forest plot of PFS comparison between two groups.

**Fig 4:**
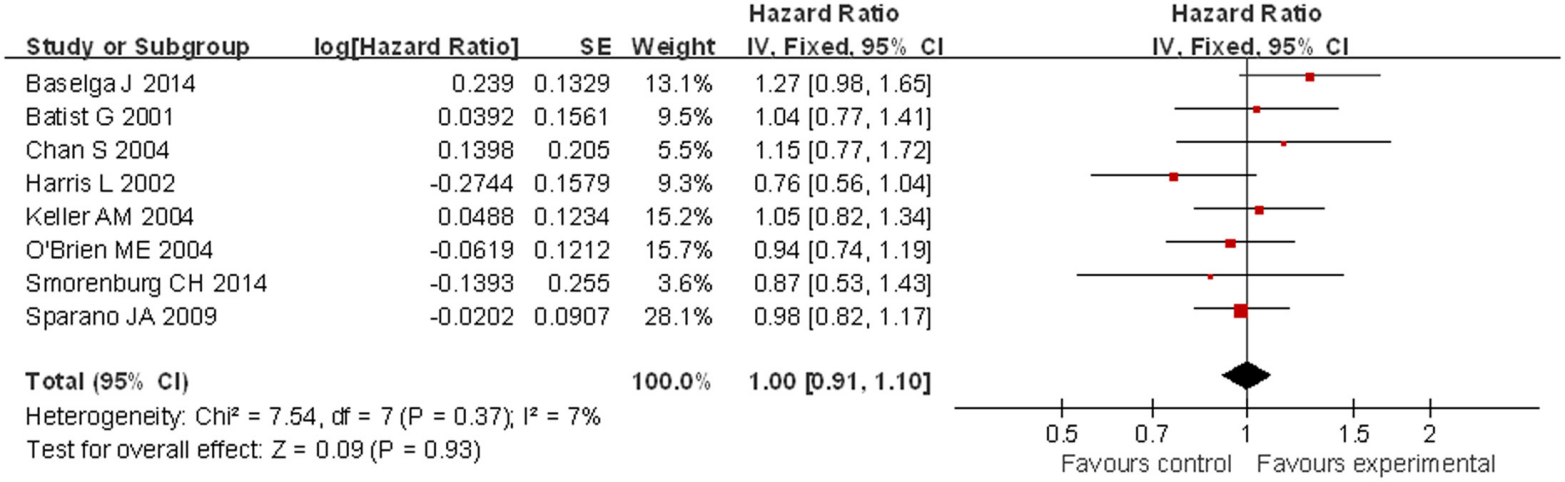
Forest plot of OS comparison between two groups.

Compared with patients treated with conventional doxorubicin (277/947), patients treated with liposomal doxorubicin-based chemotherapy (309/920) exhibited a significantly increased ORR (OR = 1.25, 95% CI 1.02 to 1.52, p = 0.03) (Fig 2). PFS was longer for patients in the liposomal doxorubicin-based group, but without a significant difference compared with the conventional doxorubicin group (HR = 1.14, 95% CI 0.96 to 1.34, p = 0.12) (Fig 3). For OS, no significant difference was noted between the liposomal doxorubicin-based group and the liposomal doxorubicin-free group (HR = 1.00, 95% CI 0.91 to 1.10, p = 0.93) (Fig 4).

### Cardiotoxicity of liposomal doxorubicin-based chemotherapy versus other chemotherapies

**Fig 5:**
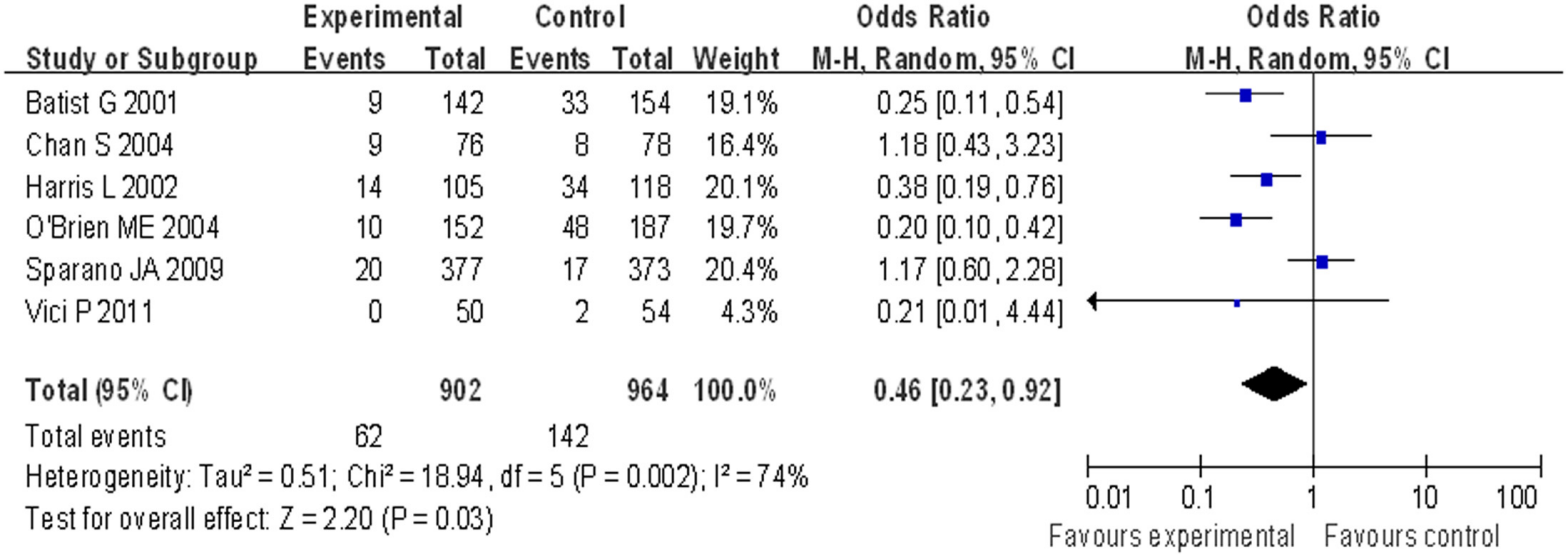
Forest plot of cardiotoxicity comparison between two groups.

Different types of chemotherapy can cause varied adverse effects. We mainly focused on cardiotoxicity, so we compared the occurrence of cardiotoxicity between groups receiving liposomal doxorubicin-based chemotherapy or conventional doxorubicin. Due to the high heterogeneity (I^2^ = 74%, p = 0.002), a random-effects model was used for determining cardiotoxicity. Compared with patients treated with conventional doxorubicin, cardiotoxicity appeared to occur less frequently in patients treated with liposomal doxorubicin-based chemotherapy (OR = 0.46, 95% CI 0.23 to 0.92, p = 0.03) (Fig 5).

## Discussion

Chemotherapy is the main option for many advanced beast cancer patients. Given that advanced breast cancer is incurable, disease control and adverse effects should be well balanced during chemotherapy. Doxorubicin is a conventional anthracycline that is highly effective in the treatment of breast cancer [23]. However, doxorubicin-associated toxicity, especially cardiotoxicity [24], limits its application. To overcome this issue, liposomal doxorubicin has been designed to reduce the cardiotoxicity of doxorubicin while preserving its antitumor efficacy [6]. In animal models, various trials have proven that the same dose of liposomal doxorubicin was associated with significantly reduced cardiotoxicity [25]. Many clinical trials also have confirmed the efficacy and cardiac safety of liposomal doxorubicin in various settings: a monotherapy or in combination with other drugs, a first-line therapy (compared with conventional doxorubicin) [19], a second-line therapy or later in patients with anthracycline- and taxane-pretreated disease (compared with vinorelbine or mitomycin/vinblastine) [18], an adjuvant therapy for older women with endocrine non-responsive disease (compared with metronomic cyclophosphamide + methotrexate) [26], or a maintenance therapy for patients with responding or stable disease after first-line chemotherapy [27]. However, there is no consensus for the superiority of liposomal doxorubicin-based chemotherapy compared with conventional doxorubicin. We conducted this meta-analysis by pooling the results of existing randomized controlled trials comparing liposomal doxorubicin-based chemotherapy with conventional doxorubicin in advanced breast cancer to draw a conclusion.

After pooling the results of existing randomized controlled trials, a statistically significant difference was observed in the ORR. For PFS, liposomal doxorubicin-based chemotherapy exhibited an apparent improvement, but a statistically significant difference was not achieved. The lack of a significant difference might be due to the relative short follow-up time. Moreover, our analysis revealed no significant difference in OS between the two chemotherapy regimens. It should be noted that advanced breast cancer patients exhibit relatively longer survivals compared with other cancer patients and receive treatments after the failure of first- or second-line chemotherapy, which will unavoidably influence the results.

After pooling the results, less cardiotoxicity was observed in patients treated with liposomal doxorubicin-based chemotherapy.

Although some reviews [6,28] have compared the efficacy and adverse effects of liposomal doxorubicin-based chemotherapy with conventional doxorubicin, our analysis is the first meta-analysis to our knowledge that combined the results of existing studies and offered more practical results. Thus, our study has reduced the effect of publication bias. Moreover, the results are encouraging. Liposomal doxorubicin may serve as a viable alternative for advanced breast cancer patients.

We acknowledge that this meta-analysis has several limitations. First, our results were based on unadjusted ORs and HRs or involved hormonal, prior-anthracycline and HER2 status. Second, the regimens in each group were not the same, which may have influenced the results. Third, with the exception of cardiotoxicity, additional adverse effects were not analyzed in our study. Fourth, the definition of cardiotoxicity based on significant left ventricular ejection fraction (LVEF) changes was not uniform across all trials.

In conclusion, liposomal doxorubicin-based chemotherapy offers significant advantages regarding the ORR and reduced cardiotoxicity relative to conventional doxorubicin in advanced breast cancer patients. For PFS and OS, future studies are needed to confirm the benefit.

## Supporting Information

S1 Checklist PRISMA Checklist. (DOC)

S1 Table Methodological quality of the included studies based on the 12-items scoring system. (DOC)

S2 Table Characteristics of the Studies Included in this Meta-analysis. (DOC)
