# Supplementary material for: Efficacy and Cardiotoxicity of Liposomal Doxorubicin-Based Chemotherapy in Advanced Breast Cancer: A Meta-Analysis of Ten Randomized Controlled Trials
Source: PLoS One. 2015 Jul 23;10(7):e0133569. doi: 10.1371/journal.pone.0133569 (PMC4512701; doi:10.1371/journal.pone.0133569)
Supplement: S1 Table — (DOC) [file pone.0133569.s002.doc]

**Table 1. Methodological quality of the included studies based on the 12-items scoring system.**

| Author | Randomised adequately^a^ | Allocation concealed | Patient blinded | Care provider blinded | Outcome assessor blinded | Acceptable drop-out rate^b^ | ITT analysis^c^ | Avoided selective reporting | Similar baseline | Similar or avoided cofactor | Patient compliance^d^ | Similar timing | Quality^e^ |
| --- | --- | --- | --- | --- | --- | --- | --- | --- | --- | --- | --- | --- | --- |
| Batist G | Yes | No | No | No | No | Yes | Yes | Yes | Yes | Yes | Yes | Yes | High |
| Harris L | Yes | No | No | No | No | Yes | Yes | Yes | Yes | Yes | Yes | Yes | High |
| Chan S | Yes | No | No | No | No | Yes | Yes | Yes | Yes | Yes | Yes | Yes | High |
| Keller AM | Yes | No | No | No | No | Yes | Yes | Yes | Yes | Yes | Yes | Yes | High |
| O’Brien ME | Yes | No | No | No | No | Yes | Yes | Yes | Yes | Yes | Yes | Yes | High |
| [Sparano JA](http://www.ncbi.nlm.nih.gov/pubmed?term=Sparano JA[Author]&cauthor=true&cauthor_uid=19687336) | Yes | No | No | No | Yes | Yes | Yes | Yes | Yes | Yes | Yes | Yes | High |
| Yardley DA | Yes | No | No | No | No | Yes | Yes | Yes | Yes | Yes | Yes | Yes | High |
| Vici P | Yes | No | No | No | No | Yes | Yes | Yes | Yes | Yes | Yes | Yes | High |
| Baselga J | Yes | No | No | No | No | Yes | Yes | Yes | Yes | Yes | Yes | Yes | High |
| Smorenburg CH | Yes | No | No | No | No | Yes | Yes | Yes | Yes | Yes | Yes | Yes | High |

^a^ Only if the method of sequence made was explicitly introduced could get a ‘‘Yes’’; sequence generated by ‘‘Dates of Admission’’ or ‘‘Patients Number’’ receive a ‘‘No’’. ^b^ Drop-out rate <20% could get a ‘‘Yes’’, otherwise ‘‘No’’. ^c^ ITT = intention-to-treat, only if all randomised participants were analysed in the group they were allocated to could receive a ‘‘Yes’’. ^d^ More than 75% patients wore respective devices for at least 3 weeks means ‘‘Yes’’, otherwise ‘‘No’’. ^e^ ‘‘Yes’’ items more than 7 means ‘‘High’’; more than 4 but no more than 7 means ‘‘Moderate’’; no more than 4 means ‘‘Low’’.
