# Supplementary material for: Efficacy and Cardiotoxicity of Liposomal Doxorubicin-Based Chemotherapy in Advanced Breast Cancer: A Meta-Analysis of Ten Randomized Controlled Trials
Source: PLoS One. 2015 Jul 23;10(7):e0133569. doi: 10.1371/journal.pone.0133569 (PMC4512701; doi:10.1371/journal.pone.0133569)
Supplement: S2 Table — (DOC) [file pone.0133569.s003.doc]

| Author | Year | Trial phase | Patient characteristics | Line of treatment | Regimens | No. of patients | Main outcomes |
| --- | --- | --- | --- | --- | --- | --- | --- |
| Batist G | 2001 | III | Stage IV | 1st | LD + CTX | 142 | Cardiotoxicity (LVEF), PFS, OS, toxicities |
|  |  |  |  |  | ADR + CTX | 155 |  |
| Harris L | 2002 | III | Stage IV | 1st | LD | 108 | ORR, PFS, toxicities |
|  |  |  |  |  | ADR | 116 |  |
| Chan S | 2004 | III | Stage IV | 1st | LD + CTX | 80 | ORR, PFS, OS, LVEF, toxicities |
|  |  |  |  |  | EPI + CTX | 80 |  |
| Keller AM | 2004 | III | Stages IIIB and IV  taxane-refractory | 2nd  or 3rd | PLD | 150 | PFS, OS, ORR, RD, EFS, tolerability, HQL, grade 3/4 toxicities |
|  |  |  |  |  | VNB/ mitomycin C + VNB | 151 |  |
| O’Brien ME | 2004 | III | Stage IV | 1st | PLD | 254 | PFS, OS, cardiotoxicity (LVEF), grade 3/4 toxicities |
|  |  |  |  |  | ADR | 255 |  |
| [Sparano JA](http://www.ncbi.nlm.nih.gov/pubmed?term=Sparano JA[Author]&cauthor=true&cauthor_uid=19687336) | 2009 | III | Stage IV | U.R. | Docetaxel | 373 | TTP, OS, ORR, cardiotoxicity (LVEF), toxicities |
|  |  |  |  |  | Docetaxel + PLD | 378 |  |
| Yardley DA | 2009 | II | Stage IV | 1st | LD | 50 | ORR, PFS, grade 3/4 toxicities |
|  |  |  |  |  | docetaxel | 52 |  |
| Vici P | 2011 | II | Anthracycline-  naive  relapsed | 1st | EPI/VNB | 50 | ORR, grade 3/4 toxicities  PFS, OS |
|  |  |  |  |  | PLD/VNB | 54 |  |
| Baselga J | 2014 | III | Stage IV  HER2 positive | 1st | NPLD + T + P | 181 | PFS, OS, toxicities |
|  |  |  |  |  | T + P | 182 |  |
| Smorenburg CH | 2014 | III | Stage IV  ≧65 years old | 1st | PLD | 40 | PFS, OS, grade 3/4 toxicities |
|  |  |  |  |  | Capecitabine | 38 |  |

U.R.: un-reported; LD: liposomal doxorubicin; CTX: cyclophosphamide; ADR: adriamycin; EPI: epirubicin; PLD: pegylated-liposomal doxorubicin; VNB: vinorelbine; NPLD: non-pegylated liposomal doxorubicin; T: trastuzumab; P: paclitaxel; PFS: PFS; OS: OS; ORR: ORR; RD: response duration; EFS: event-free survival; HQL: health-related quality of life; TTP: time to progression.
